# Determining the effective factors in predicting diet adherence using an intelligent model

**DOI:** 10.1038/s41598-022-16680-8

**Published:** 2022-07-19

**Authors:** Hediye Mousavi, Majid Karandish, Amir Jamshidnezhad, Ali Mohammad Hadianfard

**Affiliations:** 1 Department of Health Information Technology, School of Allied Medical Science, Ahvaz Jundishapur University of Medical Sciences, Ahvaz, Iran; 2 Nutrition and Metabolic Diseases Research Center, Clinical Sciences Research Institute, Ahvaz Jundishapur University of Medical Sciences, Ahvaz, Iran

**Keywords:** Health care, Nutrition, Information technology

## Abstract

Adhering to a healthy diet plays an essential role in preventing many nutrition-related diseases, such as obesity, diabetes, high blood pressure, and other cardiovascular diseases. This study aimed to predict adherence to the prescribed diets using a hybrid model of artificial neural networks (ANNs) and the genetic algorithm (GA). In this study, 26 factors affecting diet adherence were modeled using ANN and GA(ANGA). A dataset of 1528 patients, including 1116 females and 412 males, referred to a private clinic was applied. SPSS Ver.25 and MATLAB toolbox 2017 were employed to make the model and analyze the data. The results showed that the accuracy of the proposed ANN and ANGA models for predicting diet adherence was 93.22% and 93.51%, respectively. Also, the Pearson coefficient showed a significant relationship among the factors. The developed model showed the proper performance for predicting adherence to the diet. Moreover, the most effective factors were selected using GA. Some important factors that affect diet adherence include the duration of the marriage, the reason for referring to the clinic, weight, body mass index (BMI), weight satisfaction, lunch and dinner times, and sleep time. Therefore, applying the proposed model can help dietitians identify people who need more support to adhere to the diet.

## Introduction

A healthy diet includes a variety of foods and beverages that assist a person in maintaining a healthy weight, being healthy, and preventing diseases^1,2^. Diet is also an important aspect of lifestyle and personal habits that affect body composition and weight, as well as the prevention of obesity^3,4^. To prevent the complications and consequences of poor nutrition, in addition to choosing a healthy diet, it is necessary to pay attention to its continuity and adherence^3,4,6^. The concept of “adherence to diet” expresses the patient's right to choose whether or not to follow dietary recommendations and indicates the extent to which the patient actively engages in diet and treatment^5^. Many factors, such as culture and available food resources in the community, play a role in whether or not people adopt healthy diets^6^. In addition, food prices might be one of the barriers to choosing and adhering to a balanced diet, particularly among people with low income^7^. Therefore, these factors may force people to have an unhealthy diet despite their desire^8^. For example, Naalweh et al. found that adherence to diet and regimen therapy was less than the desired level in patients with dialysis, so 45% of them had low and moderate adherence to the recommended diets^9^. In contrast, a Nigerian cross-sectional survey showed that 67.4 percent of diabetic patients followed the diets^10^. Although the Mediterranean diet is consistently suggested as a healthy diet^11–14^, Daniela et al. discovered that adherence to the diet was low (16.7% of the participants aged 6–16 years).

Numerous factors, such as behavioral characteristics and lifestyle, may play a role in adherence to a diet^15^. A few additional factors, including age, ethnicity, place of residence, education, occupation, and smoking, were also discussed by Hatthachote et al.^16^. A similar study conducted in China evaluated the same parameters^17^. Another study indicated that the factors influencing adherence to diet were age, gender, education, physician–patient relationship, and social support^10^. In addition, understanding the relationship between adherence to a diet and patient education was also emphasized^9^.

As mentioned above, numerous studies have been conducted on a diet to prevent diseases and the factors affecting diet adherence^9,10,15–23^. However, these studies did not note intelligent algorithms such as neural networks and genetic algorithms to determine the influential factors in diet adherence.

Artificial neural networks have been utilized as auxiliary and standard models in classification, pattern recognition, and disease prediction^24^. Neural networks are comprised of input layers (to receive data from the user), output layers (to generate the desired responses), and hidden layers (processing layers)^25^. For example, neural networks have been used to predict people's weight after dietary interventions and following dietary recommendations^26^. Furthermore, by using machine learning methods such as KNN (K-nearest neighbors algorithm), it is possible to improve people's eating habits and ultimately control chronic diseases related to eating habits^27^.

Additionally, a genetic algorithm (GA) is an optimization and feature selection technique that can be utilized to identify relevant and effective elements^28^. This method generates a set of random solutions (individuals), where each has a different set of attributes (chromosomes). According to the rules of genetics, crossover and mutation are formed in chromosomes to develop the second generation of individuals with more diversified characteristics than the first. This procedure continues until the algorithm's termination condition is identified and satisfied. For example, It is possible to provide a subset of suitable factors for predicting diseases and nutrition-related problems when dealing with large data sets such as diet data^29^.

The importance of this study was the use of artificial intelligence in modeling behavioral patterns that may be effective in following the diet. These patterns assist dietitians in guiding patients to achieve their goals. Therefore, the present study aimed to find suitable factors to predict diet adherence using intelligent algorithms.

## Methods

In this applied study, ANN and GA were employed to predict adherence to the diet based on lifestyle factors. Although this was not an interventional or clinical trial study, all methods were carried out under the relevant guidelines and regulations and the study protocol with the reference number of IR.AJUMS.REC.1399.437 was approved by the Ethics Committee of Ahvaz University of Medical Science as well as all methods were performed in accordance with the relevant guidelines and regulations.

The data were gathered under confidentiality and privacy rules from 1528 records of a nutrition and diet therapy clinic in Ahvaz (a metropolis located in the southwest of Iran) between 2017 and 2019. Inclusion criteria comprised the record of people referred to the clinic to gain or lose weight. The records of patients with diabetes, endocrine problems, and pregnant women were excluded from the study. Criteria for adhering to the diet were considered as follows: 1. following at least three sessions of diet therapy. 2. Gaining or losing (based on the goal) 5% of the initial weight within six months^30,31^.

Twenty-six predictor variables were selected to apply in the model, including age (years), gender (male/female), education (illiterate, primary school, middle, and high education), occupation (government employed, employed in the private sector, housewife, non-employed, etc.), marital status (single/ married), duration of being married (1–5 years, 15–30 years, 30–45 years, 45–60 years, over 60 years), the reason of referring to the clinic (losing weight due to overweight or obesity, and gaining weight due to thin or very thin), clients’ body image (obese, overweight, fit, thin and very thin), Weight, height, BMI (< 18.5 underweight, 18.5–24.9 normal, 25–29.9 overweight, and >  = 30 obese), smoking, history of previous diet, weight satisfaction, history of obesity in childhood, Physical activity, having breakfast, the meal that consumes the most food, wake up time in the morning, breakfast time, lunch time, dinner time, Eating speed, sleeping time ( at night), being invited to a restaurant and party in a month, family (mother, father, sister, and brother) history of diabetes.

The variables were considered based on the literature and were selected based on availability in the patients' records. The Data were extracted from a questionnaire with 26 multiple-choice and short answer questions that patients filled out at the time of admission. Therefore, each question was measured as a variable of the model.

The current study used a feedforward net neural network of backpropagation as a classification algorithm of neural networks. After several experiments (it means several neural networks were performed to obtain the best results.), an optimized architecture including 26 input layers (number of variables of the study) and three hidden layers was generated. The number of hidden layers was usually determined based on the trial and error rule and the results’ accuracy. Therefore, the numerous hidden layers were selected, but the accuracy obtained from three hidden layers with [15 12 12] Neurons was the best. In addition, two output layers were selected based on the output results of the dependent variable. The output layers demonstrate if the individuals follow the diet or not. Therefore, the neural network model's accuracy can show the model's ability to predict diet adherence. It means that each patient, based on the questionnaire's response, was divided into one of two weight-changed and weight-not-changed groups by the model. The patients who gained the ideal weight and followed the diet belonged to the weight-changed group, and those who did not follow the diet and did not achieve the desired weight belonged to the weight-not-changed group. After entering the information of individuals in Matlab software, each group is assigned a number.

**Figure 1:**
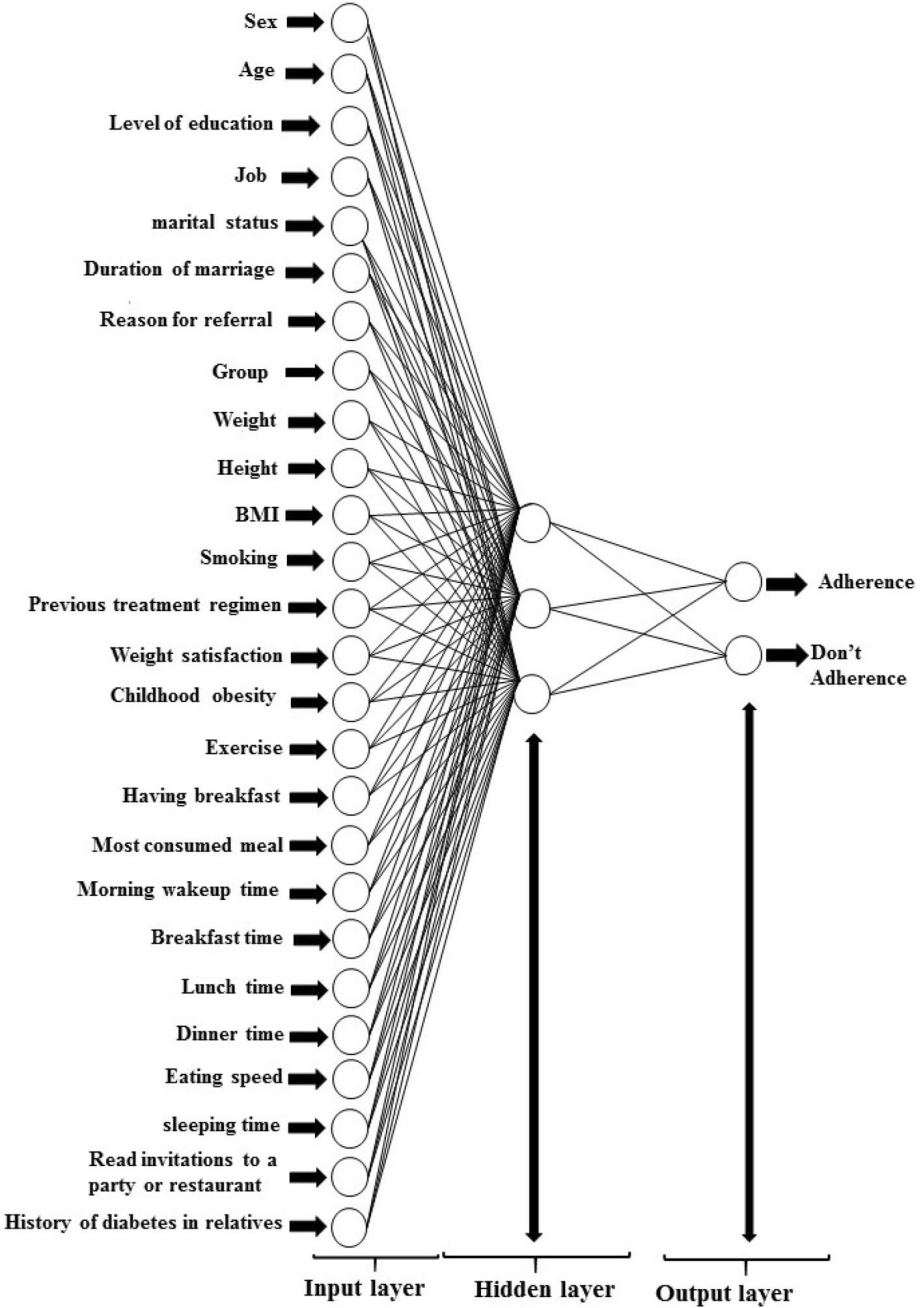
Structure of the neural network for predicting diet adherence in the patients referring to the nutrition clinic.

In this study, to continue the function training process, improve the results, and obtain optimal results in errors and weights, max_fail was considered zero. As a result, the data was divided into train and test data, with 85% and 15%, respectively. The model's sensitivity, specificity, and accuracy were calculated to measure the operating characteristics. Figure 1 depicts a sample of the neural network that was created. The trainbar (Bayesian regularization backpropagation) function was used to train the model, and the error was calculated using the Mean Squared Error (MSE) function. Also, the network was implemented using Matlab2017.

**Figure 2:**
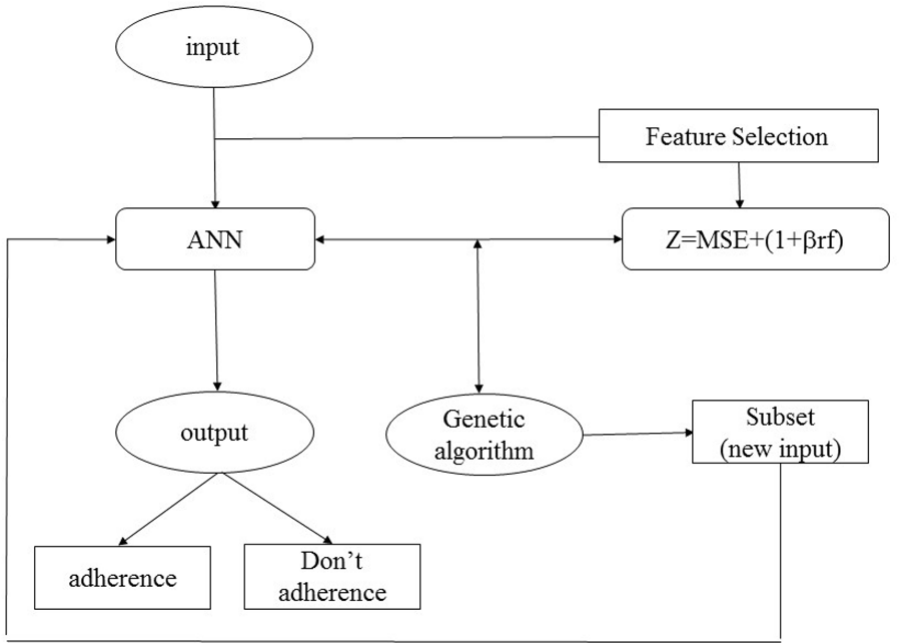
Implementation of GA for feature selection and developing proper factors for adherence to the diet.

The purpose of implementing the genetic algorithm in the study was to select a subset of the studied features with an unknown number of independent factors (26 in total) so that by choosing new factors, the MSE of the neural network is kept at a minimum, on the other hand, because the number of selected features affected the MSE, the number of selected features should also be minimized. Thus, the objective function was defined as Z = MSE + (1 + Brf). B is a coefficient of the number of selected features. The feature selection problem was coded using the Feature Selection function in conjunction with the neural network. The hybrid function was recalled as an input function of the GA to return the proper factor indices. With the selected factors as a new input in the neural network, the sensitivity, specificity, and accuracy of the neural network performance were calculated using confusion matrices compared to the neural network model with 26 inputs, which was used as a baseline. The flowchart (Fig. 2) depicts the procedure of GA implementation.

The data were analyzed using SPSS, Version 25. The percentage and frequency of all variables were obtained first in the study. Then the Pearson / Spearman coefficient was used to show an association between the variables. The significant level was determined to be 0.05. Finally, the sensitivity, specificity, and accuracy were calculated to measure the model's characteristics.

## Results

Table 1 shows that the proportion of females to males was 2.7. Most participants who followed the diets were 20 to 35 years. The education level of most participants was higher than the diploma, and only 1% were illiterate. More than half of the participants were unemployed, mainly homemakers or students. According to BMI data, more than half of the individuals were obese. In total, only 40 individuals had a history of smoking. The percentage of dissatisfaction with weight in the participants was 97.3 vs. 2.7. Only one-third of them had a history of childhood obesity. The proportion of the physical activity vs. those with no physical activity was 14.2 to 85.8, and more than half of the individuals had lunch.

**Table 1:** Basic characteristics of the prediction of continuity in referral to nutrition for diet follow-up Data presented by (number (%)).

Variables		Adherence to diet (n, %)	Do not adherence to diet (n, %)	Total (n, %)
Sex (N = 1528)	Male	217 (27.0)	195(26.9)	412(27.0)
	Female	586 (73.0)	530(73.1)	1116(73.0)
Age (N = 1528)	5–20	86(10.7)	102(14.1)	188(12.3)
	20–35	387(48.2)	351(48.4)	738(43.8)
	35–50	232(28.9)	212(29.2)	444(29.1)
	50–65	90(11.2)	53(7.3)	143(9.4)
	65–80	8(1.0)	7(1.0)	15(1.0)
Level of education (N = 1528)	Literacy	1(0.1)	1(0.1)	2(1.0)
	Elementary	22(2.7)	27(3.7)	49(3.2)
	High school	109(13.6)	99(13.7)	208(13.6)
	Diploma	223(27.8)	160(22.1)	383(25.1)
	Associate degree	52(6.5)	43(5.9)	95(6.2)
	Bachelor's degree and higher	396(49.3)	395(54.5)	791(51.8)
Job (N = 1528)	Government jobs	144(17.9)	134(18.5)	278(18.2)
	Private jobs	104(13.0)	101(13.9)	205(13.4)
	Educational jobs	50(6.2)	59(8.1)	109(7.1)
	Therapeutic occupations	18(2.2)	22(3.0)	40(2.6)
	Unemployed	487(60.6)	409(56.4)	896(58.6)
Marital status(N = 1528)	Married	502(62.5)	438(60.4)	940(61.5)
	Single	301(37.5)	287(39.6)	588(38.5)
Duration of marriage (year)	0	301(37.5)	287(36.6)	588(38.5)
	1–15	275(34.2)	292(40.3)	567(37.1)
	15–30	155(19.3)	92(12.7)	247(16.2)
	30–45	65(8.1)	47(6.5)	112(7.3)
	45–60	7(0.9)	7(1.0)	14(0.9)
Reason for referral (N = 1528)	Diet modification	9(1.1)	10(1.4)	19(1.2)
	Gaining weight	30(3.7)	98(13.5)	128(8.4)
	Fitness	1(0.1)	16(2.2)	17(1.1)
	Weight Loss	756(94.1)	487(67.2)	1243(81.3)
	High weight	7(0.9)	114(14.7)	121(7.9)
**Group (N = 1528)**				
From the patients' point of view	Overweight	426(53.1)	382(52.7)	808(52.9)
	Obese	332(43.1)	221(30.5)	553(36.2)
	Very thin	11(1.4)	33(4.6)	44(2.9)
	Thin	20(2.5)	64(8.8)	84(5.5)
	Fit	14(1.7)	25(3.4)	39(2.6)
Weight (kg)	23–65	45(5.6)	124(17.1)	169(11.1)
	63–103	571(71.1)	484(66.8)	1055(69.0)
	103–143	172(21.4)	110(15.2)	282(18.2)
	≥ 143	15(1.9)	7(1.0)	22(1.4)
Height (centimeter)	124–144	4(0.5)	8(1.1)	12(0.8)
	144–164	406(50.6)	375(51.7)	781(51.1)
	164–183	371(46.2)	329(45.4)	700(45.8)
	≥ 183	22(2.7)	13(1.8)	35(2.3)
$BMI = \frac{kg}{{\left( m \right)^{2} }}$	Underweight	19(2.4)	48(6.6)	67(4.4)
	Normal weight	34(4.2)	81(11.2)	115(7.5)
	Overweight	160(19.9)	184(25.4)	344(22.5)
	Obese	590(73.5)	412(56.8)	1002(65.6)
Smoking (N = 1528)	Yes	16(2.0)	24(3.3)	40(2.6)
	No	787(98.0)	701(96.7)	1488(97.4)
Previous treatment regimen	Yes	414(51.6)	379(52.3)	793(51.9)
	No	389(48.4)	346(47.7)	735(48.1)
Weight satisfaction	Yes	13(1.6)	28(3.9)	41(2.7)
	No	790(98.4)	697(96.1)	1487(97.3)
Childhood obesity	Yes	196(24.4)	169(23.3)	365(23.9)
	No	607(75.6)	556(76.7)	1163(76.1)
Exercise	Yes	100(12.5)	117(16.1)	217(14.2)
	No	703(87.5)	608(83.9)	1311(85.8)
Having breakfast	Some days	279(34.7)	286(39.4)	565(37.0)
	Most days	143(17.8)	141(19.4)	284(18.6)
	Everyday	381(47.4)	297(41.0)	678(44.4)
	Never	0(0)	1(0.1)	1(0.1)
Most consumed meal	Breakfast	50(6.2)	49(6.8)	99(6.5)
	Lunch	571(71.1)	464(64.0)	1035(67.7)
	Dinner	114(14.2)	140(19.3)	254(16.6)
	Breakfast and Lunch	10(1.2)	11(1.5)	21(1.4)
	Breakfast and Dinner	2(0.2)	2(0.3)	4(0.3)
	Lunch and Dinner	41(5.1)	46(6.3)	87(5.7)
	All three meals	15(1.9)	12(1.7)	27(1.8)
	None	0(0)	1(0.1)	1(0.1)
Morning wake-up time	3–8	365(45.5)	287(39.6)	652(42.7)
	8–13	425(52.9)	413(57.0)	838(54.8)
	13–18	13(1.6)	25(3.4)	38(2.5)
Breakfast time	0	86(10.7)	90(12.4)	176(11.5)
	5.30–10	476(59.3)	370(51.0)	846(55.4)
	10–14.30	241(30.0)	265(36.6)	506(33.1)
Lunchtime	0	5(0.6)	2(0.3)	7(0.5)
	11–15	623(77.6)	518(71.4)	1141(74.1)
	15–19	175(21.8)	205(28.3)	380(24.9)
Dinner time	0	62(7.7)	17(2.3)	79(5.2)
	16–22	448(55.8)	359(49.5)	807(52.8)
	22–3	293(36.5)	349(48.1)	642(42.0)
Eating speed	Slow	70(8.7)	78(10.8)	148(9.7)
	Medium	439(54.7)	418(57.7)	857(56.1)
	Fast	294(36.6)	229(31.6)	523(34.2)
sleeping time	21–1	337(42.0)	269(37.1)	606(39.7)
	1–5	459(57.2)	446(61.5)	905(59.2)
	≥ 5	7(0.9)	10(1.4)	17(1.1)
Read invitations to a party or restaurant	Unknown	5(0.6)	1(0.1)	6(0.4)
	Never	115(14.3)	86(11.9)	201(13.2)
	Rarely	22(2.7)	21(2.9)	43(2.8)
	Few	498(62.0)	435(60.0)	933(61.1)
	Much	146(18.2)	163(22.5)	309(20.2)
	Very much	17(2.1)	19(2.6)	36(2.4)
History of diabetes in relatives	Yes	478(59.5)	441(68.8)	919(60.1)
	No	325(40.5)	284(39.2)	609(39.9)

Table 2 demonstrates a significant relationship between the studied factors and diet adherence.

**Table 2:** Association of independent variables and dependent variables using Pearson / Spearman coefficient.

Variables	Adherence to diet or not	
	Pearson / Spearman correlation	Sig(2-tailed)
Sex (N = 1528)	0.001	0.955
Age (N = 1528)	0.064*	0.013
Level of education (N = 1528)	− 0.036	0.156
Job (N = 1528)	0.029	0.251
marital status(N = 510)	0.022	0.399
Duration of marriage (year)	0.061*	0.018
Reason for referral (N = 504)	0.052*	0.040
Group (N = 503) From the patients’ point of view	− 0.125**	< 0.001
Weight (kg)	0.167**	< 0.001
Height (centimeter)	0.030	0.246
BMI (N = 504)	0.197**	< 0.001
Smoking (N = 504)	0.041	0.107
Previous treatment regimen	0.007	0.779
Weight satisfaction	0.069**	0.007
Childhood obesity	− 0.013	0.615
Exercise	0.053*	0.039
Having breakfast	0.061*	0.018
Most consumed meal	− 0.041	0.111
Morning wake up time	0.071**	0.005
Breakfast time	− 0.038	0.136
Lunchtime	− 0.077**	0.002
Dinner time	− 0.147**	< 0.001
Eating speed	0.057*	0.025
Sleeping time	− 0.053*	0.039
Read invitations to a party or restaurant	− 0.061*	0.017
History of diabetes in relatives	0.013	0.604

*Correlation is significant at the 0.05 level (2-tailed).

**Correlation is significant at the 0.01 level (2-tailed).

The relationship among factors like age, duration of the marriage, the reason for referring to the nutrition clinic, physical activity, breakfast time, eating speed, sleeping time, being invited to parties and restaurants, and diet adherence was borderline statistically significant (*p* > 0.05).

Factors like group, weight, BMI, satisfaction, wake-up time, breakfast, lunch, and dinner time were strongly correlated with diet adherence (*p* > 0.01).

**Figure 3:**
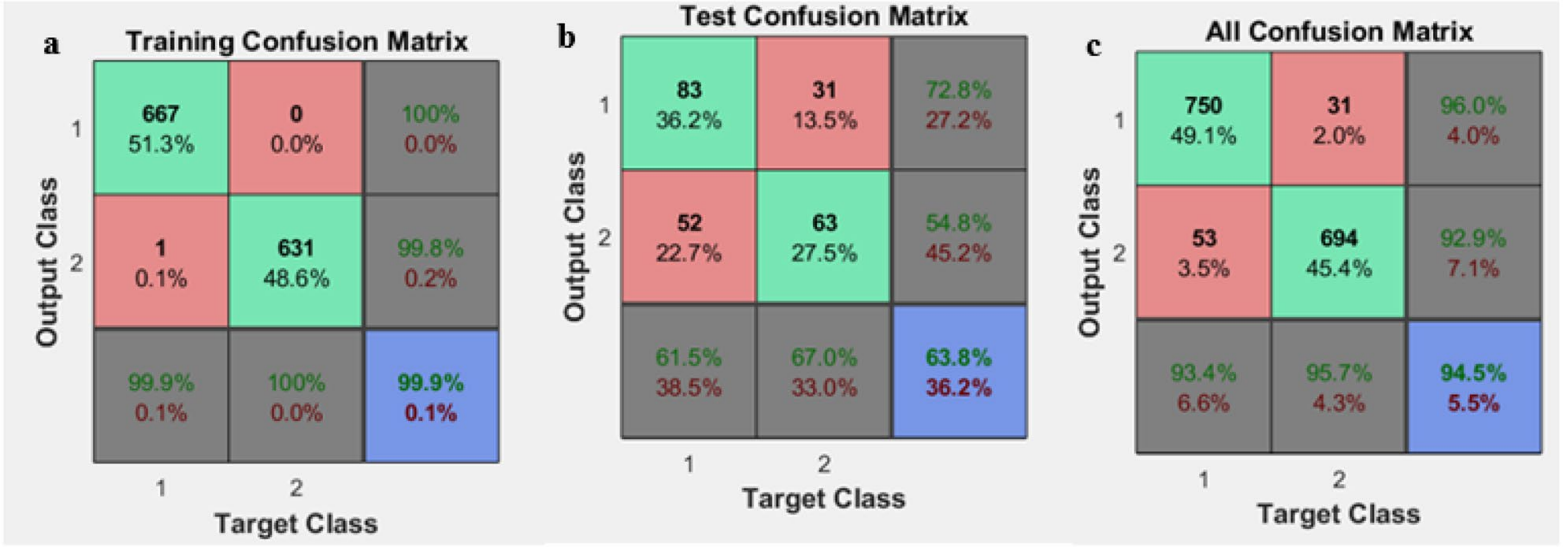
An example of confusion matrices after network implementation in traing (**a**) test (**b**) and all confusion (**c**).

After 20 times of implementing the model, the average accuracy, sensitivity, and specificity are calculated based on the confusion matrix and compared with the hybrid neural network and GA model, where its results are given in Table 3. Also, Fig. 3 shows a snapshot of the confusion matrix obtained from the neural network implementation.

**Table 3:** The results obtained from implementing the neural network before and after feature selection using GA.

Model		Sensitivity (%)	Specificity (%)	Accuracy (%)
Before Applying GA	ANN (train)	99.39	99.57	99.47
	ANN (test)	61.80	53.44	57.90
	ANN (Total)	93.82	92.60	93.22
After Applying GA	ANN (train)	98.63	99.21	98.92
	ANN (test)	61.39	56.09	59
	ANN (Total)	93.09	92.71	93.51

**Figure 4:**
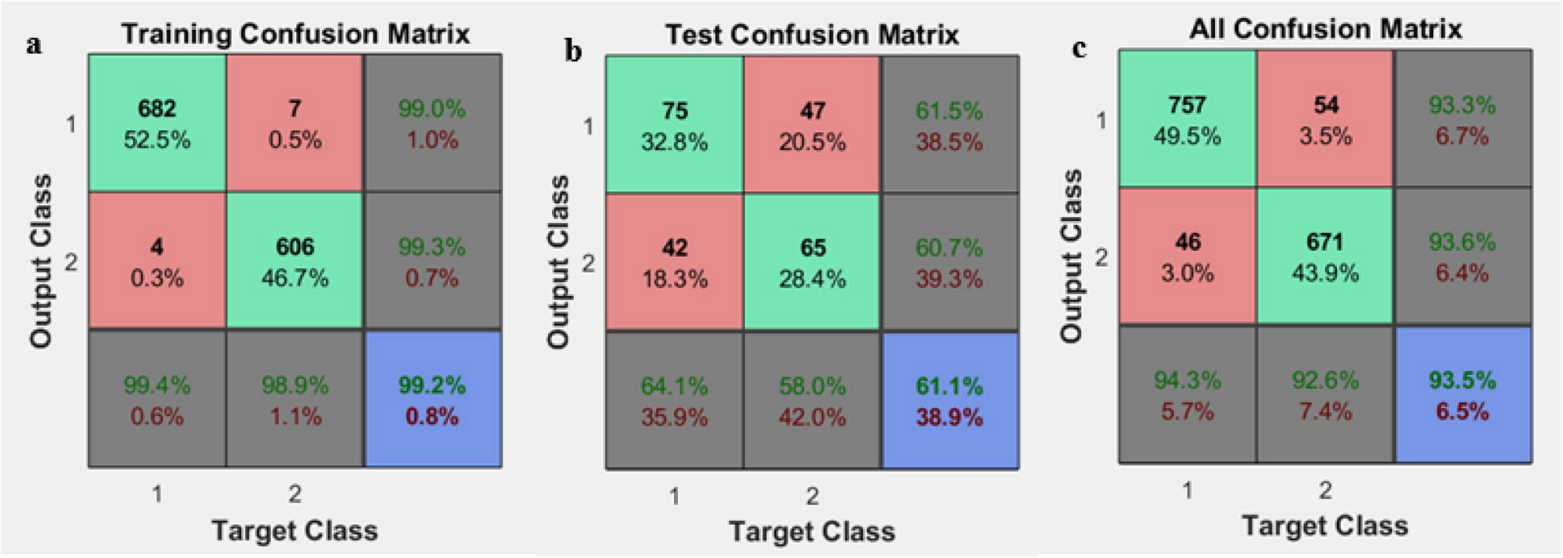
An example of confusion matrices after GA implementation in integration with ANN for selecting the effective factors. Training confusion (**a**). Test confusion (**b**) and all confusion (**c**).

**Figure 5:**
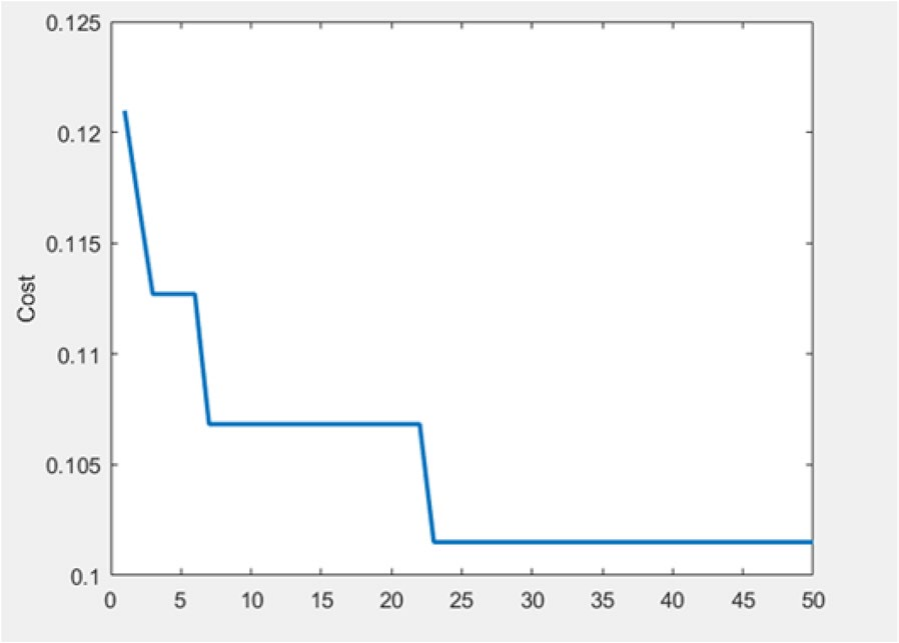
Accuracy of GA in selecting proper factors after 50 iterations.

**Figure 6:**
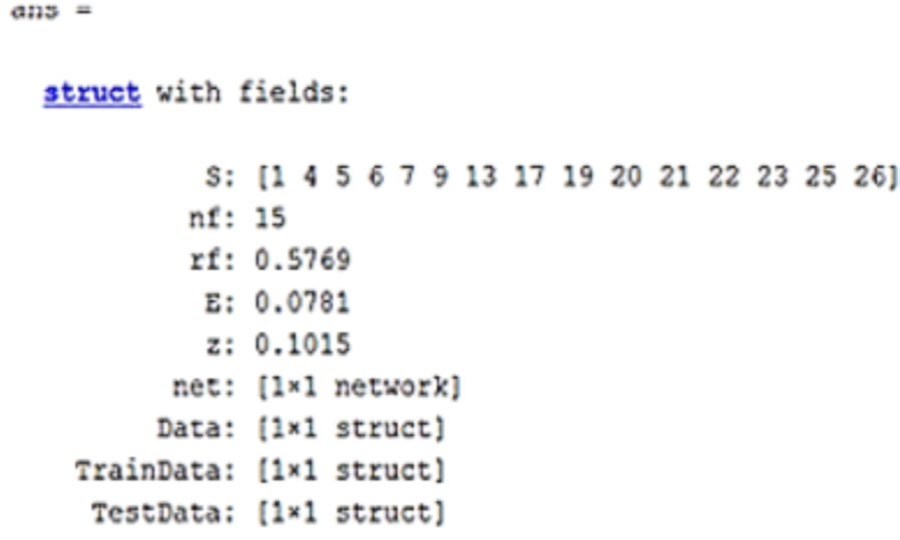
Result of implementing GA for selecting 15 effective factors in diet adherence.

GA was implemented to select proper factors in diet adherence. Due to the importance of adjusting the beta coefficient in selecting appropriate factors, beta = 0.52 was obtained to determine the suitable variables. Also, in this method, the number of iterations was 50. In this method, the initial population was 30, crossover = 0.8, and the number of offspring in each generation was calculated using the following equation:

$${\text{Nc}} = {2}*{\text{round}}\left( {{\text{pc}}*{\text{nPop}}/{2}} \right)$$

where, nc is the number of offspring in each generation, pc is the crossover percentage, and npop is the initial population. A comparable confusion matrix associated with sensitivity, specificity, and accuracy of the GA after integration with the neural network is shown in Fig. 4. In addition, a diagram of the GA's best accuracy in selecting the features affecting diet is shown in Fig. 5. The results of implementing GA showed that 15 relevant factors could be chosen to predict the continuity of the diet. The index number of these factors is shown in Fig. 6. The name of these factors is also given in Table 4.

**Table 4:** The result of performing feature selection using a genetic algorithm.

Age	1
Job	4
Marital status	5
Duration of marriage	6
Reason for referral	7
Weight	9
Previous treatment regimen	13
Having breakfast	17
Morning wake-up time	19
Breakfast time	20
Lunchtime	21
Dinner time	22
Sleeping time	23
Read invitations to a party or restaurant	25
History of diabetes in relatives	26

## Discussion

A healthy diet containing essential vitamins effectively preserves health and prevents diseases^32^. Also, avoiding high-risk factors like smoking and low physical activity^26^ might affect a healthy weight and life^33^. In addition, lifestyle changes might affect improper diet adoption^34,35^. The results showed that various factors affect diet adherence. For example, those who were overweight or underweight had a more regular continuation of their diet. Also, the study found that individuals who ate their lunch and dinner at the appropriate times of the day were more successful at sticking to their diet. Some studies showed that these factors could affect nutritional status. For example, Akbarzade et al. discovered that the volume and composition of the midday meal could impact obesity^36^. Therefore, having lunch at the wrong time will be a predisposing factor for obesity and overweight.

Furthermore, this study showed that adherence to the diet was almost equally low in both sexes, and people with a high school diploma and associated degree were more likely to adhere to the diet. Similarly, the study conducted by Morge et al. discovered that females and adults with higher education levels showed better adherence to self-care behaviors such as nutrition, exercise, and other activities^10^. In addition, the results showed that being employed could be a factor in predicting diet adherence. These results were expected because employed people have a more adjusted daily schedule than non-employed people.

Although a study in Spain showed that physical activity was effective in following the Mediterranean diet^37^, the results showed that most participants did not report exercise and physical activity. It may be due to the coronavirus and the lockdown^38^. Moreover, in line with the results of the present study, some studies showed that weight satisfaction^39^ was a significant factor in people's adherence to a diet and that individuals who were dissatisfied with their weight followed a healthier diet^40^. For instance, according to a study conducted in Georgia, body satisfaction was found to have a relationship with diet adherence. This was especially true in adolescent girls with inflammatory bowel disease, where body dissatisfaction was higher and diet adherence was lower^41^. In addition, the results showed that the participants who received many invitations to restaurants were less likely to follow their diet. This may be due to high-calorie, and unhealthy foods served in restaurants.

Furthermore, the results revealed that having a regular life plan, such as waking up in the morning at the appropriate time, going to bed at the proper time, and eating breakfast properly, were effective variables in adhering to the diet. The results of the GA model also indicated a significant relationship between the factors and adherence to the diet. To the best of our knowledge, most studies related to diet adherence have focused on the effects of lifestyle on obesity and overweight^35,42,43^. However, no study has been observed on diet adherence using intelligent methods. Although intelligent approaches such as neural networks and genetic algorithms have been utilized in many studies, no evidence has been found about diet adherence. Like the present study's intelligent method, Eduardo et al. applied an intelligent model using the MLP neural network to predict the duration and dietary changes. They implemented the model on a dataset of 105 different diets^44^. Although the present study showed that several factors could play a role in diet adherence, it did not define a suitable way to measure the degree of adherence to a proper diet. However, a study showed that awareness of a healthy diet did not improve adherence to the diet^45^.

There were several limitations in this study. First, Due to the lack of a standard, the data were collected only from the patient records of a diet clinic instead of several clinics. Second, comparing the model's accuracy with other studies was impossible since similar studies were not found. Third, whereas this study was limited to the factors obtained from the patient record, in addition to the 26 behavioral factors applied, perhaps other factors were not included in the model. Fifth, although different types of neural networks such as CNN have been used in android based applications, which have provided advice about measuring food calories^46^, a study that uses intelligent methods to predict adherence to diet has not been found.

## Conclusion

The results showed that diet adherence was significantly associated with various factors such as lifestyle, waking up early in the morning, and eating breakfast, lunch, and dinner at appropriate times. This study also showed that the proposed model using artificial neural networks had reasonable accuracy for predicting diet adherence. Also, using GA and integrating it with the neural network can increase the model's accuracy.

Applying this model can help dietitians identify patients with a low chance of diet adherence. They can employ appropriate methods such as supportive methods, for example, social networking^46^, to increase the likelihood of diet adherence and improve the effectiveness of the diet. In addition, further studies are needed to determine other factors that may affect diet adherence and provide a more comprehensive model.

## Supplementary Information

Supplementary Information.

## Data Availability

All data generated or analyzed during this study are included in this published article and its supplementary information files.
